# Current Therapies for Breast Cancer: What the Plastic Surgeon Needs to Know

**DOI:** 10.3390/jcm15187000

**Published:** 2026-09-10

**Authors:** Erin Jadallah, Natalie McConaghy, Min-Jeong Cho, Albert Chao

**Affiliations:** Department of Plastic and Reconstructive Surgery, The Ohio State University, Columbus, OH 43210, USA; erin.jadallah@osumc.edu (E.J.); natalie.mcconaghy@osumc.edu (N.M.); min-jeong.cho@osumc.edu (M.-J.C.)

**Keywords:** breast reconstruction, adjuvant therapy, reconstructive timing

## Abstract

Breast reconstruction after mastectomy or breast-conserving surgery requires careful planning that accounts for patient preference, comorbidities, tumor characteristics, and anticipated neoadjuvant or adjuvant therapies. Advances in the treatment of breast cancer have led to evolving recommendations for these therapies, with each influencing not just the complications from surgery but also the long-term outcomes. This narrative review summarizes the current indications for these therapies and their potential impacts on breast reconstruction. Contemporary recommendations for postmastectomy radiation therapy, chemotherapy, immunotherapy, endocrine therapy, and other targeted therapies are explored, with an emphasis placed on perioperative management of systemic therapies and how they may influence reconstructive decision-making. Understanding the nuances of these therapies enables plastic surgeons to be strategic in the timing of reconstruction through individualized planning and can improve reconstructive success. As breast cancer treatment evolves, familiarity with oncologic management is essential for achieving safe, patient-centered care.

## 1. Introduction

Many breast cancer patients who undergo surgery for the treatment of breast cancer will be treated through a multimodal approach, which may include radiation therapy (RT), chemotherapy, or other systemic interventions to reduce their risk of recurrence and improve overall survival (OS). Recommendations for adjuvant therapies continue to evolve as research and technologies improve, and these therapies have a direct impact on postmastectomy reconstruction timelines and outcomes. Systemic therapies and postmastectomy radiation therapy (PMRT) are associated with higher rates of delayed wound healing, infection, or reconstructive failure [1]. Emergence of novel targeted therapies and immunotherapies can potentially impact immune function and wound healing [2]. Initiating adjuvant therapies after tissue expanders (TEs) have been placed poses questions about whether TEs can or should be filled concurrently with adjuvant chemotherapy, as well as the optimal timing of their exchange with permanent implants if RT is recommended. When considering delayed–immediate reconstruction, adjuvant therapies can prolong the time until patients undergo definitive autologous reconstruction. Understanding the nuances of adjuvant therapies enables plastic surgeons to be strategic in the timing of reconstruction to improve both survival outcomes and reconstructive success. This narrative review aims to outline the current non-surgical modalities used to treat breast cancer and the criteria for each to simplify the decision-making process and better prepare reconstructive surgeons to anticipate changes in therapy needs.

## 2. Literature Search Strategy

A literature search was conducted using the PubMed database to identify relevant articles discussing breast cancer therapies and how they relate to surgery and breast reconstruction. Search terms included combinations of the following keywords: breast cancer, indications, radiation therapy, chemotherapy, immunotherapy, targeted therapy, endocrine therapy, perioperative recommendations, breast reconstruction, and reconstructive surgery. The PubMed database was searched between January 2026 and May 2026, and the most recent and clinically relevant publications were reviewed and summarized.

## 3. Current Indications for Radiation Therapy

PMRT is a useful treatment modality to reduce the risk of recurrence and improve OS for some patients with breast cancer [3]. A taskforce comprising the American Society for Radiation Oncology (ASTRO), the American Society of Clinical Oncology (ASCO), and the Society of Surgical Oncology (SSO) developed clinical practice guidelines to assess PMRT based on technique advancements and better diagnostic imaging and reduce the incidence of axillary lymph node dissection (ALND) [4]. Compared with the 2016 recommendations, the 2025 guidelines expands the scenarios in which PMRT is indicated, including recommending PMRT for patients who underwent mastectomy and ALND as their initial treatment and were diagnosed with one to three positive lymph nodes, as opposed to the prior conditional-only recommendation. PMRT can be omitted in these patients if the nodal involvement is micro-metastatic and the tumor has other favorable characteristics, such as a small tumor size or is hormone receptor (HR)-positive, or if patients are postmenopausal [4,5]. The guidelines recommend PMRT for patients with known nodal involvement, or those with multiple high-risk features (locally advanced disease, tumors ≥ 5 cm, patients under 40 years old, ER-negative, PR-negative disease). Conversely, there is a conditional recommendation for patients with a larger tumor size that is not locally advanced and in the absence of nodal involvement, or with other favorable characteristics. There is also a conditional recommendation for PMRT for those who present with limited nodal involvement and have no residual nodal disease after neoadjuvant chemotherapy. PMRT is recommended regardless of pathologic response in patients with more extensive node involvement at initial presentation. PMRT may also be recommended for patients with positive margins if re-excision is not possible [4].

For patients undergoing breast-conserving surgery (BCS) who are older than 65 years with hormone receptor-positive, early-stage breast cancer being treated with adjuvant endocrine therapy, the PRIME II trial showed that whole-breast radiation may be omitted. Although associated with higher rates of local recurrence, there was no significant difference in OS in this population [6]. For most other patients who undergo BCS, whole-breast radiation is likely to be recommended. Patients should be counseled on the potential impact on the cosmetic outcome of oncoplastic reconstruction if this is the case, and surgeons should take this into account when performing oncoplastic reconstruction and concurrent contralateral reduction. Harfouche et al. performed a retrospective review of women who underwent oncoplastic reconstruction with concurrent contralateral symmetry procedures followed by whole-breast radiation, and found that there was a 9% volume loss in the radiated breast observed at three years postop [7]. A conservative approach to oncoplastic reconstruction is to stage the contralateral side to at least six months after completion of RT. This allows for the effects of radiation to take effect and achieve better symmetry with the contralateral breast. This practice has fallen out of favor as it requires two separate surgeries and recovery periods for patients. In cases where oncoplastic breast reconstruction was not available to the patient either due to access or comorbidities, surgical delay procedures may be utilized to mitigate the risk of nipple necrosis in the radiated breast. In patients with a smaller native breast size who desire reconstruction of the lumpectomy defect with a pedicled flap, such as a thoracodorsal artery perforator (TDAP), lateral intercostal artery perforator (LICAP), or latissimus dorsi myocutaneous (LDMC) flap, it may be preferable to perform the flap in a delayed fashion rather than radiate the flap to avoid volume loss, fat necrosis, and fibrosis.

For patients with TEs who are recommended to receive adjuvant chemotherapy and radiation, the optimal timing of their exchange with permanent implants has been long debated with no clear consensus. Cordeiro et al. compared outcomes between patients who underwent RT before and after TEs were exchanged with permanent implants. They found that patients who underwent exchange after RT had a higher incidence of reconstructive failure [8]. Magill et al. performed a meta-analysis of the effects of PMRT on implant-based reconstruction, and found that radiating a permanent implant is associated with a significantly higher risk of developing capsular contracture, reconstructive failure, and the need for additional revisional surgery [9]. This lack of consensus highlights how this decision must remain patient-centered, with risks presented to guide decision-making. When attempting to exchange before RT, it is critical to coordinate the timing with radiation and medical oncologists to balance restoration of blood counts after adjuvant chemotherapy when applicable, allowing for sufficient postoperative healing before radiation starts.

The timing of free autologous reconstruction when radiation is recommended also lacks consensus among plastic surgeons. A meta-analysis performed by Heiman et al. showed significantly higher rates of flap failure in the delayed setting (average time between RT completion and surgery was 4 to 20 weeks), likely secondary to radiation effects of the internal mammaries. On the other hand, patients who underwent immediate reconstruction with subsequent RT experienced higher rates of wound healing complications. The rates of revisions and other complications, such as fat necrosis or hematoma/seroma, were similar between the two groups [10]. Liew et al. performed a meta-analysis evaluating outcomes of PMRT on immediate microvascular reconstruction and found a significantly higher risk of developing fat necrosis and volume loss compared to nonirradiated patients; however, patient satisfaction and rates of reoperation were comparable [11]. Studies suggest that if delayed autologous reconstruction is recommended, it is safest to delay for at least 6 months after completion of RT, as there is no significant benefit to delaying beyond that [12]. When considering delayed–immediate autologous reconstruction, Parmeshwar et al. found significantly higher rates of complications in the group who had their TEs radiated compared to those who underwent immediate autologous reconstruction, and they recommend the latter to mitigate risks and delays in cancer therapies [13]. Table 1 provides a summary of the current indications for radiation therapy for breast cancer according to different clinical scenarios.

## 4. Current Indications for Chemotherapy

Adjuvant chemotherapy is generally recommended for patients who are node-positive, ER-negative, or PR-negative, as chemotherapy reduces the risk of recurrence and mortality [15]. The Oncotype DX breast recurrence score was developed to provide guidance on treating estrogen receptor (ER)-positive and human epidermal growth factor receptor 2 (HER2)-negative breast cancer with adjuvant chemotherapy [16]. Based on a 21-gene assay, a score from zero to 100 is provided, with low scores defined as 0–10 and high scores defined as 26–100 [16]. Though useful, the Oncotype DX score cannot be utilized alone to determine if chemotherapy is recommended. The Trial Assigning Individualized Options for Treatment (TAILORx) evaluated the benefit of chemotherapy for patients with HR-positive or HER2-negative breast cancer with no nodal involvement and mid-range scores of 11–25. It found that there was no significant difference in the recurrence risk and OS in those who received hormone therapy (tamoxifen) and chemotherapy versus those who received hormone therapy alone, unless patients were aged 50 years old or younger with recurrence scores of 16–25 [17]. The trial RX for Positive Node, Endocrine Responsive Breast Cancer (RxPONDER) evaluated patients with HR-positive or HER2-negative breast cancer with 1–3 positive lymph nodes and oncotype scores of 0–25. There was no significant benefit of chemotherapy in the postmenopausal patients already receiving endocrine therapy. In premenopausal patients, there was an improved invasive disease-free survival in those who also received chemotherapy, even if their oncotype scores were low (0–25) [18].

There are a few standard regimens of chemotherapy, typically consisting of cyclophosphamide (C), adriamycin (A), and a taxane (T). A typical treatment plan begins with AC given every 3 weeks for four cycles, followed by T either every 3 weeks for four cycles or weekly for 12 cycles—a protocol often referred to as AC-T. Alternatives to this exist, such as administering AC-T every 2 weeks along with a growth factor [16]. The duration of these treatments can last three to five months.

Tissue expansion can be safely performed during adjuvant chemotherapy, and most surgeons recommend doing this on the day of chemotherapy treatments when blood counts are at their highest. Patients may be filled once every three to four weeks, depending on their chemotherapy schedules. If a patient is recommended to also receive radiation, and the decision is made to exchange the TEs for permanent implants prior to the start of radiation, it is recommended to wait three to four weeks after completing chemotherapy to overcome the neutropenic window [19]. In patients who receive adjuvant chemotherapy, Lee et al. demonstrated a significant increase in risks, including capsular contracture, infection, and reconstructive failure, when compared to patients who did not receive adjuvant chemotherapy. This study was adjusted for patients who also received adjuvant RT, meaning that adjuvant chemotherapy was an independent risk factor [20].

Neoadjuvant chemotherapy is utilized not only to debulk tumors preoperatively but to obtain a complete pathologic response and de-escalate the need for ALND in those with known metastatic disease of the lymph nodes. Neoadjuvant chemotherapy is generally recommended for triple-negative types, as well as HER2-positive types. Regimens for neoadjuvant chemotherapy are similar to the adjuvant setting [21]. For patients receiving neoadjuvant chemotherapy, the American Board of Breast Surgery (ASBS) recommends that patients undergo their surgeries within 3–8 weeks after completing chemotherapy to reduce risk of recurrence and improve OS [22]. Neoadjuvant chemotherapy has been shown to increase the risk of fat necrosis for autologous reconstruction, specifically for regimens that administer a taxane first [23]. A meta-analysis and systematic review completed by Nag et al. showed no significant differences in the complications or reconstructive failures in neoadjuvant chemotherapy groups versus those who did not receive neoadjuvant chemotherapy for all types of reconstruction [24].

In summary, patients with known nodal involvement after mastectomy will be recommended to receive adjuvant chemotherapy, except where the Oncotype DX score is <31, in which case, adjuvant chemotherapy may be omitted. In either case, PMRT is likely to be recommended, and knowing whether adjuvant chemotherapy is recommended will inform the timing of tissue expansion and definitive reconstruction. When adjuvant chemotherapy is not recommended, expanding and exchanging TEs for implants or performing delayed–immediate reconstruction in this timeframe is difficult and often not feasible. When adjuvant chemotherapy is recommended, patients are afforded a span of three to five months with which they may proceed with tissue expansion if they desire. This interval allows the reconstructive surgeon to exchange TEs for permanent prostheses prior to the start of radiation or to defer subsequent procedures, such as the placement of permanent prostheses or autologous reconstruction, until several months after radiation is completed.

## 5. Current Indications for Targeted Therapies and Immunotherapy

Anti-human epidermal growth factor receptor 2 (HER2)-targeted therapies suppress the signaling pathways that promote proliferation and survival in cells expressing the HER2 receptor. These can result in adverse effects of non-cancer cells that also express the HER2 receptor, most notably increasing the risk of cardiotoxicity, pulmonary toxicity, and/or hepatotoxicity [2]. According to the U.S. Food and Drug Administration, several randomized controlled trials have shown that the risk of venous thromboembolism (VTE) was higher in patients who received trastuzumab (Herceptin) and chemotherapy compared to those who received chemotherapy alone, and that VTE prophylaxis may be recommended [25]. There has been limited evidence that trastuzumab in combination with pertuzumab can impair wound healing, though clinical data have been insufficient to establish an increase in surgical complications. The ASBS’s recommendation is to continue trastuzumab and pertuzumab during the perioperative period [22].

Antibody–Drug Conjugates (ADCs), such as ado-trastuzumab emtansine (T-DM1, Kadcyla) and fam-trastuzumab deruxtecan (T-DXd, Enhertu), bind to HER2 in HER2-positive tumor cells and are internalized, releasing cytotoxic metabolites leading to cell death. Galuia et al. recommended holding these drugs for 4 weeks preoperatively and suggested that therapy can be resumed at 3–4 weeks postoperatively, largely because patients who had undergone surgery within 4 weeks of initiation of this therapy were excluded from clinical trials [26]. These recommendations were not based on direct evidence demonstrating delayed wound healing, so recommendations regarding perioperative holding of these medications should be made in combination with the medical oncologist.

Cyclin-dependent kinase 4/6 (CDK4/6) inhibitors, such as Abemaciclib (Verzenio), Ribociclib (Kisquali), and Palbociclib (Ibrance), work to slow down cancer cell reproduction and are specifically utilized to treat ER-positive, PR-positive, HER2-negative advanced or metastatic breast cancer [26]. Abemaciclib and Ribociclib are also indicated for the treatment of HR-positive or HER2-negative early breast cancer in patients who are node-positive [26,27]. The current literature has not demonstrated an increased risk of wound healing complications with Palbociclib and Ribociclib, though Galuia et al. has recommended holding these drugs for 7 days prior to surgery on a theoretical basis due to neutropenia observed when taking these drugs. Their recommendation is to resume therapy around 3 weeks postoperatively when wounds are better healed [26]. Although abemaciclib is associated with a lower risk of neutropenia, it is similarly recommended to hold this drug for 7 days prior to surgery and resume them around 14 days postoperatively [26]. These recommendations are more precautionary in their approach instead of being driven by comparative surgical outcomes. Additionally, an observational cohort study conducted by Watson et al. confirmed an increased risk of VTE for patients taking a CDK4/6 inhibitor, supporting the Food and Drug Administration (FDA)’s warning regarding the risk of VTE [28].

ADCs that target trophoblast cell-surface antigen 2 (TROP-2) are bound to chemotherapy agents and aid their entry directly into cancer cells, allowing for targeted treatment of cells over-expressing the anti-TROP-2 antigen. One of these drugs is approved in the treatment of patients with breast cancer, Sacituzumab govitecan-hziy (Trodelvy), and is approved for use in patients with locally advanced, unresectable, or metastatic breast cancer. Adverse effects of Trodelvy include diarrhea and neutropenia [2].

Immunotherapy utilizes medications that enable the immune system to fight cancer cells. Because the targets they attack may be shared with other non-cancer cells, immunotherapy can affect their function as well, making it important for surgeons to understand the optimal timing of reconstruction for patients receiving therapies that can harm healthy non-cancer cells [2]. Tumors expressing programmed death-ligand 1 (PD-L1) can bind to the programmed death 1 (PD-1) receptor on T cells, suppressing the immune response. Pembrolizumab (Keytruda) is an anti-PD-1 immune checkpoint inhibitor (ICI) [22] that blocks this signaling pathway from occurring, enhancing the immune system’s response against tumor cells expressing PD-L1 [2]. Keytruda is indicated for some patients with triple-negative breast cancer [2]. Like anti-HER2 drugs, Keytruda’s adverse reactions can include pulmonary toxicity and hepatotoxicity, and additionally can lead to nephrotoxicity, colitis, hypothyroidism, and increased risk of VTE [25]. The available clinical data have not demonstrated an increase in wound complications with perioperative pembrolizumab, and current ASBS guidance supports continuation in the perioperative period. Preoperative assessment should include evaluation for adrenal insufficiency [22]. There are currently no FDA-approved ICIs to treat HER2-positive breast cancer [29].

Much of the available evidence regarding perioperative management of systemic breast cancer therapies is limited due to the exclusion of surgical patients from clinical trials, small sample sizes, and the retrospective nature of studies. This leaves many recommendations to be based on theoretical considerations, and the differences in these recommendations make it imperative for plastic surgeons to make shared decisions with oncologists. Table 2 provides the perioperative recommendations for targeted therapies and immunotherapies.

## 6. Indications for Endocrine Therapy

Endocrine therapy is typically recommended to be taken for 5 years following cancer resection in patients with HR-positive breast cancer [30]. These therapies can include selective estrogen receptor modulators (SERMs), such as tamoxifen, for patients who are premenopausal, or aromatase inhibitors (AIs), such as anastrozole, for those who are postmenopausal or high-risk premenopausal [22,30]. Evidence for the use of neoadjuvant endocrine therapy in postmenopausal patients with HR-positive and HER2-negative breast cancers is growing, and it may be considered in certain patients to downstage tumors, potentially making breast-conserving surgery possible rather than mastectomy. For patients who are not candidates for or would prefer to avoid chemotherapy, this may also provide an alternative treatment option [30,31]. A lack of estrogens can have side effects, including decreased skin thickness, hydration, and elasticity, which can lead to wound healing complications postoperatively [32]. The clinical significance of these effects remains unclear as it relates to breast reconstruction, and there is limited data regarding the impact AIs can have on surgical outcomes.

Society guidance on perioperative tamoxifen use is mixed. The Society for Perioperative Assessment and Quality Improvement (SPAQI) supports continuing SERMs perioperatively but notes it may be reasonable to hold a SERM for about 7 days preoperatively if the patient has additional high-risk venous thromboembolism (VTE) factors [33]. The ASBS recommends a stronger precaution to hold tamoxifen for 7 days preoperatively, even if patients have no additional VTE risk factors, or for up to 3 weeks if multiple risk factors are present [22]. Systematic reviews and single-center studies report conflicting findings, with some showing no increased flap or thrombotic complications when tamoxifen is continued [34], while others report a higher incidence of flap loss or wound healing issues and recommend holding tamoxifen for 14–28 days [35,36]. Verbat et al. performed a systematic review and meta-analysis comparing the outcomes of patients using SERMs and AIs who underwent microvascular breast reconstruction and found no difference in flap loss, fat necrosis, or thrombosis of the pedicle. Their analysis did reveal an increased risk of systemic VTE with SERMs, but this did not impact reconstructive outcomes [37]. The conflicting evidence underscores the importance of individualized practice. Plastic surgeons should weigh patient VTE risk, reconstructive complexity, and oncologist input when determining whether to continue or temporarily hold tamoxifen. A summary of recommendations for perioperative management of hormone and targeted therapies is provided in Table 3.

## 7. Discussion

Formulating a plan for breast reconstruction after mastectomy requires careful consideration of many patient factors, including patient preference, body habitus, and overall health status. The introduction of adjuvant therapies can further complicate decision-making, as they impact the complication rates, aesthetic outcomes, and timing of reconstruction. Adjuvant therapy recommendations continue to evolve, as evidenced by the new recommendation for PMRT in patients who are found to have even one positive lymph node after ALND [4]. When considering the timing of reconstruction, the main area of uncertainty lies in when to recommend that TEs are exchanged for permanent implants when PMRT is recommended. Several publications have reviewed this scenario, but there is a lack of quality evidence to directly compare implant exchange that takes place prior to the initiation of and after the completion of PMRT.

Another important area that lacks consensus is perioperative management of systemic therapies, as current recommendations are based on limited evidence and expert consensus rather than prospective surgical outcomes. Clinical trials often exclude surgical patients and, therefore, do not evaluate the effects on wound healing, VTE risk, or other surgical complications. Similarly, existing data do not demonstrate a clinically significant risk of wound healing complications with the use of ICIs. Future research should evaluate the impact on breast reconstruction outcomes. Until stronger data emerge, plastic surgeons must rely on an individualized and multidisciplinary approach. Other areas for future research include the impact of interruptions in therapy around surgery on RR and OS, and if perioperative management should differ based on the type of reconstruction.

Although PubMed provided a broad database of the clinically relevant literature, publications from other databases may have been excluded. The available literature is limited by its retrospective nature and small sample size, and prospective multi-center research would be needed to establish more definitive recommendations. Amidst this complex and evolving landscape, having a sound understanding of indicated cancer therapies can guide discussions with patients and better prepare both the patient and the surgeon for the postoperative journey. It also enables plastic surgeons to better communicate with the multidisciplinary teams to improve oncologic and reconstructive outcomes.

## Figures and Tables

**Table 1:** Indications for radiation therapy.

Clinical Scenario	Recommended	Conditionally Recommended	Not Recommended
After neoadjuvant systemic therapy	CT4 or cN2-3 ^1^ regardless of pathologic response ypN+ Positive margins	cT1-3N1 or cT3N0 with pathologically negative nodes (ypN0 ^2^) Patients with high-risk features	cT1-2N0 ^3^ with ypN0 ^2^
After mastectomy + ALND	≥4 positive lymph nodes	1–3 positive lymph nodes and: -Small tumor size -ER-positive; PR-positive -Postmenopausal	pT1-2N0 ^3^
After mastectomy without nodal disease	Positive margins not amenable to re-excision	Positive margins unable to be re-excised, but the tumor has other favorable characteristics pT3N0 ^4^	Negative margins

^1^ cN2-3 = extensive regional spread [14]. ^2^ ypN0 = 0 positive lymph nodes (LN) after neoadjuvant chemotherapy, 0 LN. ^3^ c/pT1-2N0 = tumor ≤ 5 cm. ^4^ pT3N0 = tumor > 5 cm, 0 LN [14].

**Table 2:** Indications for targeted therapies and immunotherapies.

Mechanism	Drugs	Recommendations	Evidence Basis	Reason for Holding (If Applicable)
Anti-HER2	Trastuzumab (Herceptin)	Consider VTE prophylaxis, OK to continue [22]	Expert opinion supported by the available literature	VTE
	Pertuzumab (Perjeta)	OK to continue [22]	Expert opinion supported by the available literature	
	Ado-trastuzumab emtansine (Kadcyla)	Hold 4 weeks prior to surgery and 3–4 weeks postoperatively [26]	Literature review with expert recommendations	Thrombocytopenia, neutropenia
	Fam-trastuzumab deruxtecan-nxki (Enhertu)	Hold 4 weeks prior to surgery and 4 weeks postoperatively [26]	Literature review with expert recommendations	Thrombocytopenia, neutropenia
	Pertuzumab, trastuzumab, and hyaluronidase-zzxf (Phesgo)	OK to continue [22]	Expert opinion supported by the available literature	
CDK 4/6 Inhibitor	Ribociclib, Palbociclib, and Abemaciclib	Hold 7 days prior, OK to resume 2–3 weeks after surgery [22,26]	Expert opinion supported by the available literature; literature review with expert recommendations	Neutropenia, wound healing, VTE [28]
Anti-TROP-2	Sacituzumab govitecan-hziy (Trodelvy)	Hold 4 weeks prior to surgery and 4 weeks postoperatively [26]	Literature review with expert recommendations	Neutropenia
Immune Checkpoint Inhibitor	Pembrolizumab (Keytruda)	OK to continue [22]	Expert opinion supported by the available literature	

**Table 3:** Perioperative recommendations for endocrine therapies.

Class	Drug Name	Recommendations	Evidence Basis	Reason for Holding (If Applicable)
Selective estrogen receptor modulators	Tamoxifen; Toremifene	Hold 7 days prior, or hold 3 weeks prior if patient has multiple RFs for VTE [22] Hold 4 weeks prior to microvascular surgery [33]	Expert opinion supported by the available literature Retrospective cohort study	VTE
Aromatase inhibitors	Letrozole; Anastrozole; Exemestane	OK to continue [22]	Expert opinion supported by the available literature	

## Data Availability

No new data were created or analyzed in this study. Data sharing is not applicable to this article.

## Abbreviations

The following abbreviations are used in this manuscript:

ADC	Antibody–Drug Conjugate
ALND	Axillary Lymph Node Dissection
AI	Aromatase Inhibitor
ASBS	American Society of Breast Surgeons
ASCO	American Society of Clinical Oncology
ASTRO	American Society for Radiation Oncology
BCS	Breast-Conserving Surgery
CDK	Cyclin-Dependent Kinase
ER	Estrogen Receptor
FDA	Food and Drug Administration
HER2	Human Epidermal Growth Factor Receptor 2
HR	Hormone Receptor
ICI	Immune Checkpoint Inhibitor
LDMC	Latissimus Dorsi Myocutaneous
LICAP	Lateral Intercostal Artery Perforator
LN	Lymph Node
PMRT	Postmastectomy Radiation Therapy
PR	Progesterone Receptor
SERM	Selective Estrogen Receptor Modulator
SSO	Society of Surgical Oncology
TE	Tissue Expander
TNBC	Triple-Negative Breast Cancer
TROP-2	Trophoblast Cell-Surface Antigen 2
VTE	Venous Thromboembolism
